# Prediction of meningioma shrinkage after cyproterone acetate cessation

**DOI:** 10.1093/noajnl/vdag164

**Published:** 2026-06-25

**Authors:** Annabelle Collin, Virginie Montalibet, Paul E Constanthin, Olivier Saut, Julien Engelhardt

**Affiliations:** Mathematics Laboratory Jean Leray, UMR 6629, Nantes University, CNRS, Nantes, France (A.C.); Inria Center, Bordeaux University, team-project Monc, Talence, France; Inria Center, Bordeaux University, team-project Monc, Talence, France; Mathematics Institute of Bordeaux, UMR 5251, Bordeaux University, CNRS, Bordeaux INP, Talence, France; Department of Neurosurgery, Bordeaux University Hospital, Bordeaux, France; Inria Center, Bordeaux University, team-project Monc, Talence, France; Mathematics Institute of Bordeaux, UMR 5251, Bordeaux University, CNRS, Bordeaux INP, Talence, France; Mathematics Institute of Bordeaux, UMR 5251, Bordeaux University, CNRS, Bordeaux INP, Talence, France; Department of Neurosurgery, Bordeaux University Hospital, Bordeaux, France

**Keywords:** cyproterone acetate, Gompertz model, meningiomas, progesterone, random forests

## Abstract

**Background:**

Progestin-induced meningiomas may stabilize or regress after discontinuation of cyproterone acetate (CPA), but the determinants of this volumetric response remain unclear. Identifying predictors of tumor shrinkage could improve patient management and prognosis.

**Methods:**

We retrospectively analyzed the longitudinal volumetric evolution of 137 meningiomas diagnosed during ongoing CPA treatment in 52 consecutive patients. Tumor volumes were measured from all available imaging studies. Four mathematical models (linear, exponential, power, and the Gompertz) were compared to describe tumor growth trajectories. Response patterns were derived from the Gompertz modeling. Predictive models were constructed using random forests, with leave-one-out cross-validation. Baseline clinical and radiological variables, as well as early volumetric response at 3 months, were evaluated as predictors.

**Results:**

The Gompertz model best described tumor volume evolution. Three patterns of tumor response after CPA withdrawal were identified: response with low limit volume (47%), response with high limit volume (41%), and non-response (12%). At the patient level, analogous clusters were found: low limit response (22%), high limit response (64%), and non-response (14%). Random forest models predicted cluster membership with error rates of 24.8% at the tumor level and 23.1% at the patient level. The most informative predictors were initial tumor burden and patient age at CPA exposure and withdrawal. Incorporating early volumetric response at 3 months reduced the prediction error to 9.5% for tumors and 3.8% for patients.

**Conclusions:**

Tumor burden and age at CPA exposure are major predictors of meningioma shrinkage after treatment discontinuation. Early volumetric changes provide additional prognostic value and significantly improve predictive accuracy.

## Graphical Abstract

**Graphical Abstract:**
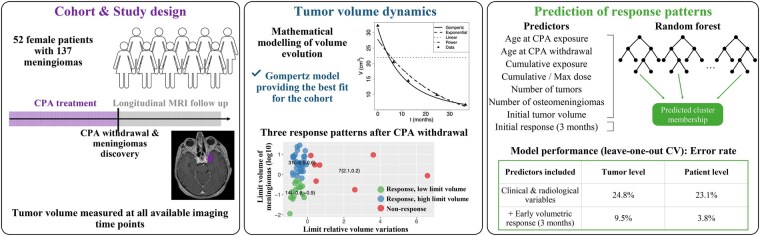

Key Points

- Tumor-level response after CPA stop: 47% low-limit, 41% high-limit, 12% none
- Initial tumor burden and age at exposure are top predictors of shrinkage.
- Early 3-month volume change cuts prediction error to 9.5% (tumors), 3.8% (patients).

Importance of the Study

Discontinuing cyproterone acetate often stabilizes or shrinks meningiomas, but clinicians lack tools to anticipate who will regress and by how much. In a longitudinal cohort of 137 tumors from 52 patients, a Gompertz model captured post-withdrawal dynamics and revealed 3 response profiles (responder with low-limit volume, responder with high-limit volume, non-responder). Random-forest analysis identified initial tumor burden and age at CPA exposure/cessation as dominant predictors, adding the 3-month volumetric change reduced error rates to 9.5% (tumor-level) and 3.8% (patient-level), enabling early risk-adapted management. Compared with prior reports centered on descriptive shrinkage frequencies, this study delivers an individualized prediction framework to guide surveillance versus intervention and to better set expectations with patients. Future implications include external validation and radiomics-based models to enable baseline-only prediction and broader deployment across centers.

Meningiomas are the most common primary intracranial tumors.^1^ Their hormonal sensitivity has been suspected since Cushing and Eisenhardt observed a substantially skewed sex ratio favoring women.^2^ Cyproterone acetate (CPA) is a synthetic progestin with progesterone receptor (PR) agonist properties and anti-androgenic action. Although it is not approved for use in the United States, it is used in Europe and Canada for various indications, including the treatment of hirsutism, acne, and endometriosis at a dose of 25 to 50 mg daily. It is also used in combination with ethinyl estradiol as an oral contraceptive at a lower dose.^3^ At the population level, high doses of CPA may increase the risk of developing meningiomas in a dose-dependent manner.^4-11^ At the tumor level, meningiomas may decrease in size after CPA withdrawal, as reported in several case series^12-35^ regression was observed in 72% of cases, according to Voormolen et al.^16^ However, the factors predicting a decrease in meningioma size after treatment discontinuation remain unknown, representing a significant gap in clinical practice. Although some meningiomas can be safely monitored in anticipation of volumetric reduction or stabilization, others may require earlier intervention because of their size or location if they do not shrink substantially or rapidly. The ability to predict the likelihood of meningioma shrinkage for an individual patient would be a valuable tool in clinical management. The aim of this study was to identify predictive factors associated with post-CPA shrinkage and to develop models capable of anticipating tumor response.

## Methods

### Study Design and Inclusion Criteria

This is a retrospective cohort study. All consecutive patients diagnosed with one or more intracranial meningiomas during ongoing CPA treatment and referred to the Department of Neurosurgery of the Bordeaux University Hospital from December 2011 to December 2020 were included. Tumors were categorized as meningiomas and osteomeningiomas based on the presence of a well-identified measurable osteoma on imaging. We included only the volumes of the meningeal components of osteomeningiomas in the analysis, as the temporal behavior of the osseous components differs.^36-38^

### Data Collection

The following data were obtained from the medical records and patients’ imaging: age at meningioma diagnosis and treatment cessation, surgical treatment and, if applicable, histology, and details of CPA treatment (dosage and monthly dosing schedule, treatment duration, and cumulative dose). Meningioma localization was described as *global* (ie anterior fossa, middle fossa and supratentorial tentorium, posterior fossa and infratentorial tentorium, anterior and posterior convexity, relative to the coronal suture, respectively, parafalcine and parasagittal) and *detailed* according to the International Consortium on Meningiomas classification.^39^ Meningiomas were also classified into 2 topographic categories based on their presumed mutational profile: posterior convexity, parafalcine, parasagittal, and posterior fossa meningiomas, which are presumed to harbor NF2 alterations and/or 22q loss, and, conversely, anterior and middle fossa and anterior convexity meningiomas, which are presumed to harbor non-NF2 alterations, including TRAF7, AKT1, KLF4, SMO, and PIK3CA mutations.^40^^,^^41^

### Control Cohort

Findings were compared with a control cohort of 333 patients exhibiting incidental meningiomas and no history of progestin use, which we previously investigated in another study.^42^ This cohort is referred to as the historical cohort (HC) in the text.

### Image Analysis

For each meningioma, at least one baseline image acquired at the time of diagnosis and corresponding to the discontinuation of CPA treatment (denoted by $t_{0}$) was available, followed by a series of longitudinal follow-up images. The number of images per meningioma ranged from 2 to 10, with an average of approximately 5 images per case. Volumes were measured from three-dimensional (3D) millimetric T1-weighted gadolinium-enhanced magnetic resonance imaging (MRI) scans. All patients were routinely monitored with contrast-enhanced 3D T1-weighted MRI, ensuring complete imaging data for the study. All images were analyzed by the senior author neurosurgeon using *Sophia Radiomics* © software, which enables 3D volumetric assessment through a semi-automatic segmentation algorithm tailored for meningiomas. We denoted by V(t) the measured tumor volume at time t.

### Fitting of Time-Volume Curves

Our first objective was to gain insight into the dynamics of the measured volumes through the use of mathematical modeling. We denoted as $\hat{V}(t)$, the estimated volume at time t, as ${\hat{V}}_{0}$ the estimated volume at first imaging $t_{0}$. The hat notation allows to differentiate the measured volumes from the estimated volumes. We considered the 4 following mathematical models for tumor volume evolution: the linear model, the exponential model, the power model, and the Gompertz model, see Supplementary Material A for more details. The model parameters were estimated using a population approach via MonolixSuite 2024R1 in R. Model selection based on AIC, BIC, and MSE identified the Gompertz model as best, see also Supplementary Material A for more details.

### Tumor Growth and Evolution Parameters

When using the Gompertz model, we defined the minimal (for shrinking tumors ie α<0) or the maximal (for growing tumors ie α>0) volume that could be theoretically reached as the time tends to infinity by the limit volume, denoted by ${\hat{V}}_{\lim}$, see Supplementary Material A for a full definition. We then defined the corresponding limit relative volume variation


$${\hat{\mathrm{RV}}}_{\lim}=\frac{{\hat{V}}_{\lim}-{\hat{V}}_{0}}{{\hat{V}}_{0}},$$

which summarizes the amplitude and speed of tumor growth or decay over time. We denoted a meningioma as *decreasing* (resp. *increasing*) if ${\hat{\mathrm{RV}}}_{\lim}<0$ (resp. ${\hat{\mathrm{RV}}}_{\lim}>0$).

Given that many patients present with multiple meningiomas exhibiting distinct evolutionary patterns, we additionally introduced the sum of limit volumes and the weighted mean of limit relative volume variations. More precisely, the limit volume sum was denoted by $\sum {\hat{V}}_{\lim}$ and was defined by


$$\sum {\hat{V}}_{\lim}=\sum_{k=1}^{N}{\left({\hat{V}}_{\lim}\right)}_{k},$$

where,

-
N is the number of meningiomas of the considered patient,
-
${\left({\hat{\mathrm{RV}}}_{\lim}\right)}_{k}\;\mathrm{is}\;\mathrm{the}\;\mathrm{theoretical}\;\mathrm{limit}\;\mathrm{volume}\;\mathrm{computed}\;\mathrm{for}\;\mathrm{the}\;k$ is the theoretical limit volume computed for the *k*-th meningioma of the patient.

Then, the weighted mean of limit relative volume variations was denoted by ${\hat{\mathrm{WRV}}}_{\lim}$ and defined by


$${\hat{\mathrm{WRV}}}_{\lim}=\frac{\sum_{k=1}^{N}{\bar{V}}_{k}{\left({\hat{\mathrm{RV}}}_{\lim}\right)}_{k}}{\sum_{k=1}^{N}{\bar{V}}_{k}},$$

where,

-
${\bar{V}}_{k}\;\mathrm{is}\;\mathrm{the}\;\mathrm{average}\;\mathrm{volume}\;\mathrm{over}\;\mathrm{time}\;\mathrm{of}\;\mathrm{the}\;k$ is the average volume over time of the *k*-th meningioma of the patient,
-
$\bar{V}$ is the average volume over time of all the tumors of the patient,
-
${\left({\hat{\mathrm{RV}}}_{\lim}\right)}_{k}\;\mathrm{is}\;\mathrm{the}\;\mathrm{theoretical}\;\mathrm{maximal}\;\mathrm{volume}\;\mathrm{decrease}\;\mathrm{computed}\;\mathrm{for}\;\mathrm{the}\;k$ is the theoretical maximal volume decrease computed for the *k*-th meningioma of the patient.

### Clustering

To better characterize the effect of CPA treatment withdrawal—both at the tumor and patient levels—and to investigate whether this response could be predicted from baseline variables, either alone or in combination with early treatment dynamics, we defined clusters based on the previously introduced metrics.

Two main criteria were used to form these clusters:

- The *limit volume* (for individual tumors) or its *sum* (for patients), representing the ultimate tumor burden,
- The *limit relative volume variation* (for tumors) or its *weighted mean* (for patients), summarizing the average shrinkage or growth tendency.

Based on these criteria, 3 clusters were defined according to 2 distinct thresholds $s_{th}^{m}$ and $s_{th}^{p}$:

- **Responder with low limit volume**: ${\hat{\mathrm{RV}}}_{\lim}<0\;\mathrm{and}\;{\hat{V}}_{\lim}<s_{th}^{m}\;\mathrm{for}\;\mathrm{tumors};\;{\hat{\mathrm{WRV}}}_{\lim}<0\;\mathrm{and}\;\sum {\hat{V}}_{\lim}<s_{th}^{p}$ for patients;
- **Responder with high-limit volume**: ${\hat{\mathrm{RV}}}_{\lim}<0\;\mathrm{and}\;{\hat{V}}_{\lim}\geq s_{th}^{m}\;\mathrm{for}\;\mathrm{tumors};\;{\hat{\mathrm{WRV}}}_{\lim}<0\;\mathrm{and}\;\sum {\hat{V}}_{\lim}\geq s_{th}^{p}$ for patients;
- **Non-responder (NR)**: ${\hat{\mathrm{RV}}}_{\lim}\geq 0\;\mathrm{for}\;\mathrm{tumors};\;{\hat{\mathrm{WRV}}}_{\lim}\geq 0$ for patients.

At tumor level, the threshold value $s_{th}^{m}$ was selected based on a hierarchical clustering procedure of the $\log_{10}$-distribution of the parameters ${\hat{V}}_{\lim}$. Visual inspection of the dendrogram initially suggested 4 clusters, corresponding to the longest branches of the tree. However, since retaining too many clusters was not deemed reasonable, we subsequently merged them pairwise based on their centroid values. At patient level, the threshold value $s_{th}^{p}$ was selected based on a hierarchical clustering procedure of the $\log_{10}$-distribution of the parameters $\sum {\hat{V}}_{\lim}$, with the dendrogram clearly revealing two distinct clusters.

### Cluster Prediction

Random forest (RF) classifiers were investigated to predict cluster membership at both the tumor and patient levels. Concerning the tumor clustering, the following baseline variables were used: detailed localization, global localization, localization by presumed driver mutation, age at treatment cessation, age at treatment initiation, cumulative dose, CPA cumulative exposure, maximal daily dose intake, number of tumors, initial tumor volume (${\hat{V}}_{0}$), sum of the initial volumes of other tumors in the same patient, and the relative tumor volume. Relative tumor volume was defined for each lesion as the ratio between the initial tumor volume and the sum of the initial volumes in the same patient. CPA cumulative exposure was computed as the ratio of the cumulative dose to a standard regimen of 50 mg/day for 21 days per month and expressed in years of equivalent exposure.

Concerning the patient clustering, the following baseline variables were used: age at treatment cessation, age at treatment initiation, cumulative dose, CPA cumulative exposure, maximal daily dose intake, number of tumors and sum of initial volumes of all the patient’s meningioma, denoted by $\sum {\hat{V}}_{0}$.

The early relative volume change (RVC_3_) $\frac{{\hat{V}}_{3\text{months}}-{\hat{V}}_{0}}{{\hat{V}}_{0}}$ (for tumors) or its sum $\frac{\sum {\hat{V}}_{3\text{months}}-\sum {\hat{V}}_{0}}{\sum {\hat{V}}_{0}}$ (for patients), where ${\hat{V}}_{3\text{months}}$ denotes the estimated volume at 3 months according to the Gompertz model, were also considered in additional models.

Missing values were imputed using the mean of the corresponding variable across available tumors, thereby ensuring inclusion of all individuals in the analysis. Given the limited sample size, a leave-one-out cross-validation strategy was applied. In addition, to account for cluster size imbalance, a weighted RF algorithm was used.

### Statistical Analyses

All analyses were performed using R software. Quantitative variables are presented as means ± standard deviations or medians (25th-75th percentiles), depending on their distribution; qualitative variables are presented as absolute values with percentages. Quantitative variables were compared between groups using the Mann-Whitney U test; the Kruskal-Wallis test was used for multiple comparisons, and the Dunn test was used for post-hoc analyses. Qualitative variables were compared using Fisher’s exact test or the $\chi^{2}$ test as appropriate. Bonferroni correction was applied for multiple pairwise comparisons. Predictive models for shrinkage clusters were developed using RF classifiers (using the implementation from R ranger library), computed with a leave-one-out strategy to prevent data leakage between the training and validation sets. To quantify uncertainty around the reported performance metrics at the patient level, 95% confidence intervals were derived using a stratified bootstrap procedure (2,000 resamples), in which observations were resampled with replacement within each cluster class separately.

## Results

### Patients and Meningiomas

During the study period, 54 consecutive women receiving CPA treatment were referred to our department after diagnosis of at least one meningioma. Of these, 4 underwent surgery immediately after diagnosis because of a mass effect and neurological impairment. Two of these patients also had additional tumors that were monitored. One additional patient with 3 meningiomas underwent resection of 2 of them after less than 2 months of follow-up because of worsening symptoms. Finally, 137 tumors (116 meningiomas and 21 osteomeningiomas) in 52 patients were included in the analysis.

Clinical, radiological, and follow-up data are provided in Table 1. A detailed analysis of tumor localization, see Supplementary Material, Supplementary Table S1, showed a significant difference between CPA-induced meningiomas (M cohort), and non-progestin induced meningiomas (HC cohort), P<.001. Specifically, CPA-induced meningiomas were more frequently located in the anterior convexity and lateral sphenoid wing as compared to non-progestin induced meningiomas (31.4% and 16.8% versus 16.9% and 6.6%, respectively, adjusted P<.05). In contrast, non-progestin-induced meningiomas were more commonly located in the falx cerebri and the posterior fossa as compared to CPA-induced meningiomas (17.8% and 4.5% versus 4.4% and 0%, respectively, adjusted P<.05). No significant differences were observed for other localizations. Additionally, the median age at meningioma diagnosis was significantly younger in the M group than in the HC (51 [46.9; 56.7] vs. 58.51 [48.95-67.88] years, respectively; P<.001). Furthermore, the volume of the meningiomas at diagnosis was significantly smaller in the M group than in the HC (0.54 [0.13-3.34] vs. 1.3 [0.43-3.37] cm${}^{3}$, respectively; P=.003).

**Table 1.** Clinical and radiological data of CPA-induced meningiomas

Patients	*N* = 52
Age at treatment start	33.5 [22.4; 40.0]
Age at treatment cessation	51 [46.9; 56.7]
Treatment length (years)	16.5 [13.8; 24.3]
Cumulative dose (g)	189 [100.8; 270]
Maximal daily dose (mg)	50 [31.3; 50]
Number of tumors in a same patient	2 [1; 3]

Meningiomas	*N* = 137
Follow-up (months)	36.5 [25.3; 53.9]
Number of observations	5 [4; 6]
$V_{0}$ (cm${}^{3}$)	0.54 [0.13; 3.29]
Surgery after observation time	8 (5.8%)
Pathology	7 Meningothelial (WHO1); 1 secretory (WHO1)
*Global localization*	
Anterior fossa	37 (27.0%)
Middle fossa and tentorium	32 (23.4%)
Posterior fossa and tentorium	1 (0.7%)
Anterior convexity	43 (31.4%)
Posterior convexity	11 (8.0%)
Parafalcine	6 (4.4%)
Parasagittal	7 (5.1%)
*Localization by presumed driver mutation profile*	
Non-NF2	112 (81.8%)
NF2	25 (18.2%)

Continuous variables are given as median [Q1; Q3].

### Meningiomas as Independent Observations

**Figure 1.**
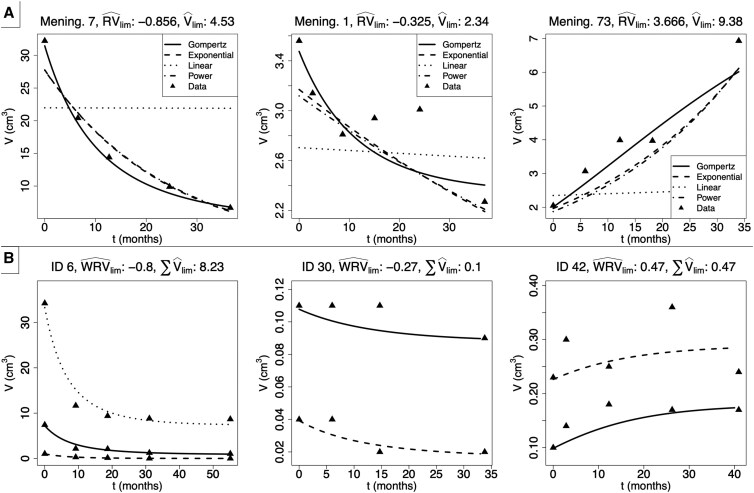
Different volumetric courses over time after CPA withdrawal. (A) Each figure represents a single meningioma, and each curve—distinguished by line type—depicts the predicted trajectory from a different model. The computation of ${\hat{\mathrm{RV}}}_{\lim}$ and ${\hat{V}}_{\lim}$ can be seen on the top of each plot. Left column: strong decrease; middle column: slight decrease; right column: sustained growth. (B) Each figure represents a single patient, and each curve—distinguished by line type—corresponds to a different meningioma fitted using the Gompertz model. The computation of ${\hat{\mathrm{WRV}}}_{\lim}$ and $\sum {\hat{V}}_{\lim}$ can be seen on the top of each plot. Left column: strong decrease; middle column: slight decrease; right column: sustained growth.

The Gompertz model provided the best fit for the data across growing and shrinking tumors with an AIC of -329 (compared to -166 for power model, -154 for exponential model and 192 for linear model). It was therefore selected for analyses of volumetric changes in what follows. Figure 1—top presents the volumetric trajectories of 3 meningiomas over time along with the corresponding fits for all models. The estimated theoretical maximal volume ${\hat{V}}_{\lim}$ (from the Gompertz model) and the associated growth rate ${\hat{\mathrm{RV}}}_{\lim}$ for each selected meningioma are indicated in the plot titles.

**Figure 2.**
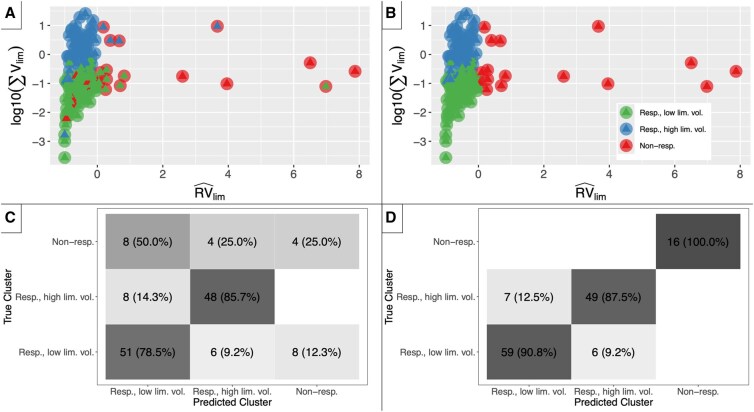
Prediction of meningioma cluster assignment response. (A) Predicted values (triangles) vs true values (circle) without the variable RVC_3_. (B) Predicted values (triangles) vs true values (circle) without the variable RVC_3_. (C) Confusion matrix without RVC_3_. (D) Confusion matrix with RVC_3_.

An illustration of the clusters is given in Figure 2—top through a scatter plot of ${\hat{\mathrm{RV}}}_{\lim}$ (x-axis) versus $\log10({\hat{V}}_{\lim})$ (y-axis), where only the circles (not the triangles) should be considered. To summarize, the clustering analysis identified the following groups:

- **Responder with low limit volume (RLLV)** (green circles): 65 meningiomas (47%), centroid at $\left({\hat{\mathrm{RV}}}_{\lim},\log_{10}({\hat{V}}_{\lim}))=(-0.6,-1.3\right)$,
- **Responder with high limit volume (RHLV)** (blue circles): 56 meningiomas (41%), centroid at $\left({\hat{\mathrm{RV}}}_{\lim},\log_{10}({\hat{V}}_{\lim}))=(-0.5,0.2\right)$,
- **NR** (red circles): 16 meningiomas (12%), centroid at $\left({\hat{\mathrm{RV}}}_{\lim},\log_{10}({\hat{V}}_{\lim}))=(2.2,-0.4\right)$.

To determine whether meningiomas that continued to grow after CPA withdrawal (ie NR) exhibited different growth dynamics relative to growing non-progestin-induced meningiomas, we compared the parameters of the Gompertz model between these 2 groups: growing meningiomas (referred to as MG) versus the non-progestin-induced meningiomas cohort (HC). The Gompertz model parameters included the intrinsic growth rate at $t_{0}$, denoted by α (in months${}^{-1}$), and the coefficient of the rate of decrease in α over time, denoted as β (in months${}^{-1}$). The analysis revealed that CPA-induced meningiomas had a higher initial growth rate compared with non-progestin-induced meningiomas (α, 0.03 [0.02-0.06] vs 0.01 [0.01-0.02], respectively, P=.015), and that their growth rate decreased more rapidly (β, 0.06 [0.04-0.07] vs 0.02 [0.01-0.04], respectively, P<.001).

**Figure 3.**
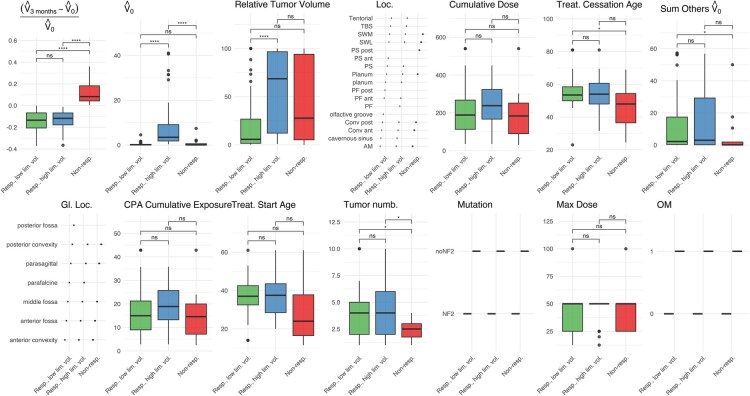
Distribution of baseline variables across meningioma clusters. Variables are sorted by normalized importance. Statistical significance of differences between clusters is indicated by asterisks: * for *P* < .05, ** for *P* < .01, *** for *P* < .001, and *ns* for non-significant comparisons.

Results of the predictive models using only baseline variables are given in Figure 2—left and in Figure 2—right when adding the variable RVC_3_. Sensitivity and specificity for cluster prediction were, respectively, 0.79/0.78 for RLLV; 0.86/0.88 for RHLV and 0.25/0.93 for NR, with a global error rate of 24.8%. Adding the early relative volume change RVC3 increased the sensitivity and specificity to 0.91/0.90 for RLLV; 0.88/0.93 for RHLV and 1.00/1.00 for NR, with a global error rate of 9.5%. The variables most used by the models for prediction were, in descending order of normalized importance: ${\hat{V}}_{0}$ (1), age at treatment initiation (0.259), age at treatment cessation (0.256), relative tumor volume (0.211), cumulative exposure (0.116), and cumulative dose (0.109). When adding RVC_3_ in the models, the variables most used by the models for prediction were, in descending order of normalized importance: RVC_3_ (1), ${\hat{V}}_{0}$ (0.683), and relative tumor volume (0.054). To facilitate interpretation, Figure 3 depicts the distribution of baseline variables across meningioma clusters, along with the results of statistical tests (*P*-value) indicating the significance of the differences.

### Patients as Independent Observations

Thirty-one (69%) patients had multiple meningiomas with a mean of around 3.4 tumors per patient. Analysis revealed that 6 patients (11.5%) had exclusively growing meningiomas (Cluster NR), 10 patients (19%) had only decreasing meningiomas from Cluster RLLV, and 11 patients (21%) had only decreasing meningiomas from Cluster RHLV. The remaining patients (48%) exhibited meningiomas with heterogeneous volumetric trajectories.

Figure 1—bottom illustrates this intra-individual heterogeneity in tumor behavior: for example, ID6 (left) shows a patient with a substantial average decrease in tumor volumes, ID30 (middle) shows a patient with a modest average decrease in tumor volumes, and ID42 (right) shows a patient with modest growth on average. Considering the sum of limit volumes of meningiomas for each patient, denoted by $\sum \hat{V}l\mathrm{im}-$ we observed that some patients had, on average, meningiomas showing a substantial decrease in volume, yet the total limit volume remained large, see eg ID6, Figure 1—bottom-5-left, whereas some of them had a modest average decrease in tumor volumes but with a small limit volume sum, see, eg ID30, Figure 1—bottom-middle.

**Figure 4.**
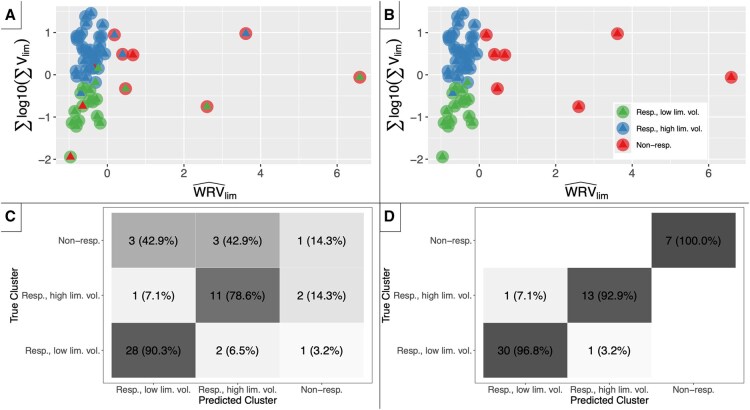
Prediction of patient cluster assignment response. (A) Predicted values (triangles) vs true values (circle) without the variable RVC_3_. (B) Predicted values (triangles) vs true values (circle) without the variable RVC_3_. (C) Confusion matrix without RVC_3_. (D) Confusion matrix with RVC_3_.

An illustration of the patient clusters is given in Figure 4 through a scatter plot of ${\hat{\mathrm{WRV}}}_{\lim}$ (x-axis) versus $\log10(\sum {\hat{V}}_{\lim})$ (y-axis), where only the circles (not the triangles) should be considered. To summarize, the clustering analysis identified the following groups:

- **Responder with low limit volume (RLLV)** (green circles): 14 patients (27%), centroid at $\left({{\hat{\mathrm{WRV}}}_{\lim},\log}_{10}(\sum {\hat{V}}_{\lim}))=(-0.6,-0.9\right)$,
- **Responder with high limit volume (RHLV)** (blue circles): 31 patients (57%), centroid at $\left({{\hat{\mathrm{WRV}}}_{\lim},\log}_{10}(\sum {\hat{V}}_{\lim}))=(-0.5,0.6\right)$,
- **NR** (red circles): 7 patients (14%), centroid at $\left({{\hat{\mathrm{WRV}}}_{\lim},\log}_{10}(\sum {\hat{V}}_{\lim}))=(2.1,0.2\right)$.

**Figure 5.**
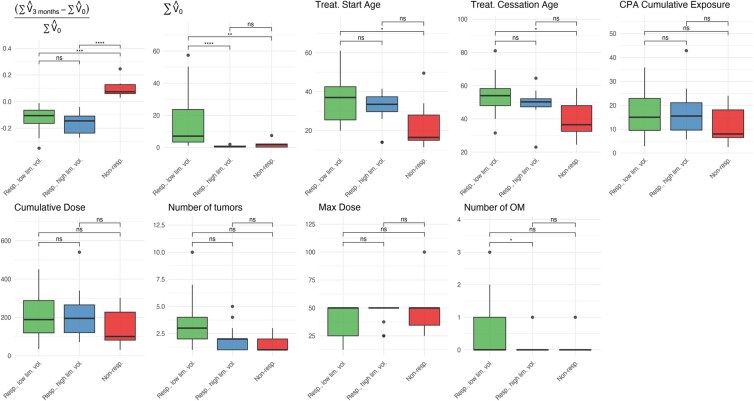
Distribution of baseline variables across patient clusters. Variables are sorted by normalized importance. Statistical significance of differences between clusters is indicated by asterisks: * for *P* < .05, ** for *P* < .01, *** for *P* < .001, and *ns* for non-significant comparisons.

Results of the predictive models using only baseline variables are given in Figure 4—left and in Figure 4—right for the case with the use of variable RVC_3_ = $\frac{\sum {\hat{V}}_{3\text{months}}-\sum {\hat{V}}_{0}}{\sum {\hat{V}}_{0}}$. Sensitivity and specificity for cluster prediction were, respectively, 0.90/0.81 for RLLV; 0.79/0.87 for RHLV and 0.14/0.93 for NR, with a global error rate of 23.1%. Adding the early relative volume change RVC3 increased the sensitivity and specificity to 0.97/0.95 for RLLV; 0.93/0.97 for RHLV and 1.00/1.00 for NR, with a global error rate of 3.8% (0%-9.6%). Stratified bootstrap confidence intervals on per-class sensitivity were (0.87-1.00) for RLLV, (0.79-1.00) for RHLV and (1.00-1.00) for NR. The variables most used by the models for prediction were, in descending order of normalized importance: $\sum {\hat{V}}_{0}$ (1), age at treatment initiation (0.380), age at treatment cessation (0.260), maximum dose (0.09), cumulative exposure (0.09) and cumulative dose (0.08). When adding RVC_3_ in the models, the variables most used by the models for prediction were, in descending order of normalized importance: RVC_3_ (1), $\sum {\hat{V}}_{0}\;$(0.839) and age at treatment initiation (0.041) To facilitate interpretation, Figure 5 illustrates the distribution of baseline variables across patient clusters, along with the results of statistical tests (*P*-value) indicating the significance of the differences.

## Discussion

This study sought to identify predictive factors of volumetric response following treatment cessation in patients with CPA-associated meningiomas and to develop mathematical models capable of anticipating tumor shrinkage both at the level of individual lesions and patients.

First, the clinical and radiological characteristics of our cohort significantly differed from those of non-progestin-induced meningomas.^42^ CPA-associated meningiomas were diagnosed at a younger age and at a smaller tumor volume. This may be related to the systematic brain MRI screening performed in patients exposed to CPA, in accordance with French regulatory recommendations. In line with this hypothesis, Samoyeau et al^5^ reported in a real-life French screening program that 76% of meningiomas detected in women receiving progestins were smaller than 1 cm. CPA-associated meningiomas were more frequently located in the anterior and middle skull base or anterior pre-coronal convexity and were more often multiple (80%) compared with only 21% in the control population.^42^ These findings are consistent with prior series^8^^,^^9^^,^  ^43^^,^^44^ and likely reflect a distinct biological profile of progestin-induced tumors, including their specific mutational spectrum. The predilection for anterior locations may relate to known anteroposterior gradients in mutational distribution,^41^ the prevalence of PIK3CA/AKT1 mutations in CPA-associated lesions,^45-47^ and embryologic differences in meningeal origin.^48^

Among the 4 growth models tested, the Gompertz model most accurately described both progression and regression dynamics. This model incorporates a time-dependent decrease in growth or regression rates, accounting for the sigmoidal pattern of growing lesions and the characteristic rapid initial shrinkage followed by a plateau observed for shrinking ones. The latter likely reflects an initial phase of regression due to sudden removal of the hormonal growth stimulus, followed by a steady-state phase once metabolic demands are balanced. Whether such tumors remain stable indefinitely or may eventually regrow remains uncertain, though no cases of regrowth after regression have been documented to date. This model allowed the observation that even CPA-associated tumors that continued to grow after CPA withdrawal exhibited higher initial growth rates but more rapid subsequent slowing compared to non-CPA meningiomas. This suggests partial sensitivity to treatment cessation, even in lesions that do not regress. Moreover, time-volume curve analysis and clustering revealed that only 12% of the tumors were classified as “non-responder,” which is much lower than the 28% observed by Voormolen et al.^16^

Beyond their value for understanding and describing tumor dynamics, mathematical models are particularly useful for defining response clusters. They allow for direct comparison of the entire tumor evolution rather than only the observed segments, and they can accommodate the highly variable intervals between patient follow-up exams. In particular, they provide estimates of the asymptotic volumes of all meningiomas even when only a few follow-up exams are available. We chose to base our clustering on these estimates considering both the limit volumes at the tumor and patient levels, as well as the relative variations in volume. These criteria are, of course, debatable, as the way clusters are defined has a major impact on the study. Our choices were guided by the idea that treatment response is best characterized by the combination of ultimate tumor burden and the tendency toward shrinkage or growth.

At both tumor and patient levels, younger age at CPA initiation and cessation were significantly associated with poorer volumetric response, suggesting reduced reversibility of growth when exposure occurs earlier in life. In contrast, cumulative exposure, cumulative dose, and maximum dose were not consistently associated with post-withdrawal shrinkage despite substantial variability in treatment regimens. Nonetheless, the RF model suggested a modest contribution of cumulative and maximum dose, indicating that dose-related factors may retain some predictive value. This association was, however, much weaker than the robust dose-dependent relationship reported in epidemiologic studies for tumor induction,^6^^,^  ^8^ suggesting that the biological mechanisms underlying tumor initiation and post-withdrawal regression may not fully overlap.

At the lesion level, larger baseline volume was associated with shrinkage with high limit volume and larger relative tumor volume was associated with shrinkage with low limit volume. Furthermore, at the patient level, higher total tumor volume was associated with shrinkage with low limit volume. This finding aligns with those of Voormolen et al^16^ who observed a significant correlation between the initial volume and the rate of tumor shrinkage. These observations may indicate that higher tumor burden increases the likelihood of CPA-induced tumors, enhancing sensitivity to hormonal withdrawal.

We did not observe any differences in shrinkage rates based on tumor location. This result is consistent with the findings of Voormolen et al^16^ who reported that tumor location was not predictive of shrinkage velocity in multivariate analysis. The hypothesis that meningioma location influences regression after CPA discontinuation may be partly mediated by its close association with driver mutations. Prior studies have shown that CPA-exposed meningiomas are enriched in PIK3CA and TRAF7 alterations and depleted in NF2 alterations, particularly at skull base locations, while NF2-mutated tumors may be less likely to shrink after withdrawal.^13^^,^  ^47^ In our cohort, this issue is further reinforced by the marked imbalance in tumor distribution, as 82% of tumors were located in presumed non-NF2 topographies. Such limited representation of NF2-alteration locations substantially reduces the ability to detect an independent topographic effect. Therefore, the absence of a significant association between location and shrinkage likely reflects the structural distribution of CPA-exposed cohorts rather than a true lack of biological relevance.

We used RF models rather than logistic regression as the primary predictive approach for several reasons. First, RF can naturally handle non-linear relationships and complex interactions between predictors, which are likely present in clinical variables such as tumor size, patient age, treatment dose, and early volumetric response. Second, RF can accommodate variables of different types (continuous, categorical, ordinal) without extensive preprocessing. Third, RF is robust to noisy variables and reduces overfitting by aggregating multiple decision trees. Fourth, it provides variable importance measures, helping to identify the most influential predictors of tumor response. Finally, unlike standard binary logistic regression, which only models 2 classes, RF can directly handle multiclass outcomes, such as the 3 tumor response clusters (low-limit, high-limit, non-response), without requiring complex transformations. For these reasons, RF was considered well suited to the objectives of the present study. We additionally explored a simpler patient-level multinomial log-linear model based on the same predictors when early response is considered. Although this model retained some predictive value, its performance was clearly lower than that of the RF approach, with a higher overall error rate 21.2% (11.5%-30.8%) versus 3.8% (0.0%-9.6%) and a markedly lower sensitivity for the NR class 0.43 (0.14-0.71) versus 1.00 (1.00-1.00). All 7 prediction disagreements between the 2 models corresponded to errors of the logistic model, not of the RF. This suggests that, despite the apparent simplicity of the dominant predictors, the association between these variables and patient response profile is not purely linear and is better captured by a more flexible modeling strategy.

Using only baseline pharmacologic and tumor data, models could accurately distinguish responders with low or high limit volumes but failed to identify NRs. The addition of early volumetric change (over the first 3 months) substantially improved classification accuracy for all response categories, underlining the clinical utility of short-term follow-up imaging. However, this approach cannot be applied to large, symptomatic tumors where observation is not feasible.

As a retrospective study, our work is subject to recall bias in pharmacologic history, mitigated through prescription verification and physician contact. Volumetric measurements are inherently prone to inter-observer variability, which we minimized by employing semi-automatic segmentation software and smoothing via mathematical modeling. In this study, volumetric contouring was not systematically subjected to a formal double expert review, which may be particularly relevant for skull base meningiomas extending into anatomically complex regions such as the cavernous sinus. To address this point, we performed an additional agreement analysis comparing the semi-automatic Sophia Radiomics © volumetric assessment used in the study with a manual re-segmentation performed by a senior neurosurgeon in 35 lesions. This analysis showed no systematic difference between methods (Wilcoxon signed-rank test, *P* = .984), excellent absolute agreement (ICC = 0.996, 95%, CI 0.992-0.998), and minimal bias on Bland-Altman analysis (mean bias, -0.066; limits of agreement, -1.125 to 0.992). Although this does not fully replace an independent double expert contouring process, these findings suggest that the volumetric endpoint was sufficiently robust and that segmentation-related variability was unlikely to have materially affected the main study conclusions.

While model error rates ranged from 3.8% to 9.5% in cross-validation, independent external validation is required before integration into routine clinical practice. A first model limitation of our approach is that the classification of patients as good or poor responders after treatment discontinuation relies on the estimation of the asymptotic tumor volume ${\hat{V}}_{\lim}$, defined in the Gompertz model as the volume reached when time tends to infinity. This parameter offers a synthetic descriptor of the long-term growth (or decay) tendency and makes clustering possible, but it is necessarily obtained through extrapolation. However, several aspects mitigate this limitation: the use of a population approach for parameter estimation, the relatively large number of available time points, and the fact that, for most meningiomas, the estimated limit volume remained reasonably close to the last observed volume, thanks to the long follow-up in our cohort. Altogether, these elements suggest that the reliance on ${\hat{V}}_{\lim}$ does not represent a major concern. A second model limitation concerns prediction. For this purpose, we chose to use both the initial tumor volume estimated by the model and, above all, the estimated volume at three months. The first was included mainly for consistency, while the second was preferred because the observed three-month volume was not available for all patients. This choice is pragmatic but may limit generalization. In future work, this limitation could be addressed by validating the approach in another cohort ideally with systematic follow-up including an exam at 3 months, for instance, in another hospital center with a 1-year monitoring protocol.

Strengths of this study include the robust sample size (137 tumors), the mean of 4.9 volumetric measurements per lesion, and a mean follow-up of 27.6 months. The use of previously validated modeling methodology and exhaustive pharmacologic data collection allowed for a detailed exploration of biological and clinical predictors, supporting the development of predictive models for tumor shrinkage after CPA cessation with good accuracy. These results provide a quantitative framework for refining follow-up strategies and personalizing management in this specific meningioma population.

## Conclusion

Tumor burden and age at CPA exposure are key predictors of shrinkage after treatment withdrawal, while early volumetric change markedly improves prediction accuracy. Although the most clinically relevant question is to identify, at diagnosis, which tumors will not regress after CPA withdrawal, our results suggest that baseline variables alone remain insufficient for fully reliable prediction. In practice, assuming limited measurement error, MRI-based volumetric assessment at baseline and at 3 months may help identify meningiomas unlikely to regress after CPA withdrawal. External validation is needed before routine clinical use, and future work should explore radiomic analysis to enable prediction from baseline diagnostic data alone.

## Supplementary Material

vdag164_Supplementary_Data

## Data Availability

All codes, along with the post-processing deidentified data (patient information, volumes, etc.), will be made available online once the article is accepted.
