# Supplementary material for: Prediction of meningioma shrinkage after cyproterone acetate cessation
Source: Neurooncol Adv. 2026 Jun 25;8(1):vdag164. doi: 10.1093/noajnl/vdag164 (PMC13368815; doi:10.1093/noajnl/vdag164)
Supplement: vdag164_Supplementary_Data [file vdag164_supplementary_data.docx]

**Supplementary Materials**

1. Models and parameter estimation

We considered the four following mathematical models for tumor volume evolution:

- Linear model:

$$\hat{V}\left( t \right)=\hat{V}_{0}+\alpha\left( t-t_{0} \right),$$

- where $\alpha$ is the slope of the growth. A positive (resp. negative) value of $\alpha$ implies a growth (resp. a decrease).
- Exponential model:

$$\hat{V}\left( t \right)=\hat{V}_{0}e^{\alpha\left( t-t_{0} \right)},$$

- corresponding to the explicit solution of the ordinary differential equation:

$$\hat{V}'=\alpha\hat{V} \text{with} \hat{V}\left( t_{0} \right)=\hat{V}_{0},$$

- where $\alpha$ is the growth/decrease rate (assumed to be constant). A positive (resp. negative) value of $\alpha$ implies a growth (resp. a decrease).
- Power model:

$$\hat{V}\left( t \right)=\left( \hat{V}_{0}^{1-\gamma}+\alpha\left( 1-\gamma\right)\left( t-t_{0} \right) \right)^{\frac{1}{1-\gamma}},$$

- corresponding to the explicit solution of the ordinary differential equation:

$$\hat{V}'=\alpha\hat{V}^{\gamma} \text{with} \hat{V}\left( t_{0} \right)=\hat{V}_{0}.$$

- This model is based on the hypothesis that the volume growth is proportional to a power – denoted by $\gamma$ – of the volume. The proportionality coefficient is denoted by $\alpha$. A positive (resp. negative) value of $\alpha$ implies a growth (resp. a decrease).
- Gompertz model:

$$\hat{V}\left( t \right)=\hat{V}_{0}\exp\left( \frac{\alpha}{\beta}\left( 1-e^{-\beta\left( t-t_{0} \right)} \right) \right),$$

- corresponding to the explicit solution of the ordinary differential equation:

$$\hat{V}'=\alpha e^{-\beta t}\hat{V} \text{with} \hat{V}\left( t_{0} \right)=\hat{V}_{0}.$$

- In this model, $\alpha$ is the initial growth/decrease rate and $\beta^{-1}$ corresponds to the characteristic time at which the tumor growth capacity decreases. At a fixed initial growth/decrease rate $\alpha$, a larger value of $\beta$ leads to a quicker growth/decrease in growth rate. A positive (resp. negative) value of $\alpha$ implies a growth (resp. a decrease).

To estimate the model parameters $\hat{V}_{0}$, $\alpha$, $\beta$, and $\gamma$, a population approach was used, as described in our previous work^42^. The only modification concerns the parameter constraints: to account for a possible decrease in volume over time, we assumed a normal distribution for the parameter $\alpha$ rather than a lognormal distribution. The parameters were estimated via MonolixSuite 2024R1 in R software (R Foundation for Statistical Computing, Vienna, Austria). Goodness of fit was evaluated using the Akaike information criterion (AIC), the Bayesian information criterion (BIC) and the mean squared error (MSE) calculated for each individual meningioma, between estimated and observed volumes. The model with the smallest average AIC over meningioma from cohort (M) was initially selected; the BIC and MSE were subsequently considered to leverage all three criteria. This procedure selected the Gompertz model.

When using the Gompertz model, we defined the minimal (for shrinking tumors *i.e.* $\alpha<0$) or the maximal (for growing tumors *i.e.* $\alpha>0$) volume that could be theoretically reached as the time tends to infinity by the limit volume

$$\hat{V}_{\lim}=\lim_{t\to\infty}\hat{V}\left( t \right)=\hat{V}_{0}e^{\frac{\alpha}{\beta}}.$$

1. Supplementary results

**Table S1. Detailed localization, with comparison with the HC cohort.**

| **Localization (ICOM)** | **M, n (%)** | **HC, n (%)** | **p (adjusted)** |
| --- | --- | --- | --- |
| anterior fossa NOS | 9 (6.6) | 9 (2.7) | 0.116 |
| cavernous sinus* | 7 (5.1) | 7 (2.1) | 0.195 |
| Convexity, anterior | 43 (31.4) | 58 (17.4) | 0.0077 |
| Convexity, posterior | 11 (8.0) | 52 (15.6) | 0.090 |
| Olfactive groove | 2 (1.5) | 2 (0.6) | 0.625 |
| parafalcine | 6 (4.4) | 59 (17.7) | 0.00085 |
| parasagittal | 7 (5.1) | 36 (10.8) | 0.116 |
| planum | 9 (6.6) | 19 (5.7) | 0.674 |
| SWL | 23 (16.8) | 22 (6.6) | 0.0077 |
| SWM | 15 (10.9) | 16 (4.8) | 0.068 |
| TBS | 3 (2.2) | 16 (4.8) | 0.410 |
| Tentorium | 2 (1.5) | 16 (4.8) | 0.188 |
| Posterior fossa | 0 (0.0) | 15 (4.5) | 0.0288 |
| Ectopic | 0 (0.0) | 3 (0.9) | 0.625 |
| Convexity NOS | 0 (0.0) | 4 (1.2) | 0.410 |

^*^Part of SWM in ICOM classification. NOS: not otherwise specified.
